# Micro-RNAs Let7e and 126 in Plasma as Markers of Metabolic Dysfunction in 10 to 12 Years Old Children

**DOI:** 10.1371/journal.pone.0128140

**Published:** 2015-06-05

**Authors:** Bernardo J. Krause, Ivo Carrasco-Wong, Angélica Dominguez, Pilar Arnaiz, Marcelo Farías, Salesa Barja, Francisco Mardones, Paola Casanello

**Affiliations:** 1 Division of Obstetrics and Gynecology, School of Medicine, Pontificia Universidad Católica de Chile, Santiago, Chile; 2 Department of Public Health, Faculty of Medicine, Pontificia Universidad Católica de Chile, Santiago, Chile; 3 Division of Pediatrics, Faculty of Medicine, Pontificia Universidad Católica de Chile, Santiago, Chile; John Hopkins University School of Medicine, UNITED STATES

## Abstract

**Background:**

Growing evidence shows that metabolic syndrome (MetS) is already starting in childhood however there is no consensus regarding how to diagnose this condition in pediatric population. Studies in adults show that altered levels of specific micro-RNAs are related with components of the MetS.

**Objective:**

We determined the plasma levels of four MetS-associated micro-RNAs (miR-126, miR-132, mir-145 and Let-7e) in 10 to 12 years old children with or without MetS traits.

**Design:**

Pediatric subjects were selected from a cohort of 3325 school-age children, and clustered by the absence (control, n = 30), or the presence of 1 (n = 50), 2 (n = 41) or 3 (n = 35) MetS traits according to Cook´s criteria. Micro-RNAs were isolated from plasma, and levels of miR-126, miR-132, miR-145 and Let-7e were determined by Taqman qPCR.

**Results:**

Regression analysis of the different MetS traits regarding the different miRNAs analyzed showed that Let-7e presented a negative association with HDL-C levels, but a positive correlation with the number of MetS traits. Levels of miR-126 presented a positive correlation with waist circumference, waist to hip ratio, BMI, and plasma triglycerides and VLDL-C. Levels of miR-132 showed a positive correlation with waist to hip ratio. Plasma levels of Let-7e were increased (~3.4 fold) in subjects with 3 MetS traits, and showed significant AUC (0.681; 95%CI = [0.58, 0.78]; p < 0.001) in the ROC analysis which were improved when miR-126 was included in the analysis (AUC 0.729; p < 0.001). *In silico* analysis of the interaction of proteins derived from mRNAs targeted by Let7 and miR-126 showed an important effect of both Let-7e and miR-126 regulating the insulin signaling pathway.

**Conclusions:**

These results suggest that changes in the plasma levels of Let-7e and miR-126 could represent early markers of metabolic dysfunction in children with MetS traits.

## Introduction

The metabolic syndrome (MetS) in adults is commonly defined as the concomitance of cardiometabolic alterations including central obesity, elevated fasting glucose, hypertriglyceridemia, low plasma HDL and arterial hypertension [1]. Due to its growing prevalence worldwide during the last decades efforts have been focused to unveil the underlying mechanisms that precede MetS. Initial studies aimed to discover the genetic basis of this disease; however evidence suggests that the MetS results from the interaction of genetic, life style, environmental and early life factors. In this context, compelling data shows that MetS risk is increased in subjects with altered fetal and early infancy growth, suggesting the premature establishment of MetS susceptibility [2].

Notably, an important increase in childhood obesity during the last years has emerged, leading to higher risk of MetS in these subjects [1]. At the present time there is no consensus whether children and adolescents could present MetS, and a clear criteria to define it in the pediatric population is lacking [1, 3]. Possible definitions have been suggested by Cook and colleagues [4], and the International Diabetes Federation (IDF) [1]which have proposed that the MetS can be considered in (1) children aged 6–10 years who present central obesity [defined as waist circumference (WC) ≥ 90^th^ percentile] and have other relevant risk factors (i.e. family history of cardiometabolic disease), and in (2) children aged 10–16 years who are obese (defined as WC ≥ 90^th^ percentile) and meet the adult metabolic syndrome criteria for triglycerides (TGs), HDL-cholesterol (HDL-C), blood pressure (BP), and glycaemia. However the consistency of these parameters used for the MetS in adults are in conflict with normal metabolic changes that take place during puberty and evade the fact that MetS risk factors are a continuum that begins with subtle alterations [3]. Altogether these data suggest the necessity for additional markers to improve the MetS diagnosis in the pediatric population.

During the last years plasma micro-RNAs (miRNAs) have emerged as potential biomarkers in diverse pathologic conditions due to their high stability in serum compared with other types of RNA [5]. Wide-screening studies in adult blood samples show that despite the presence of hundreds of circulating miRNAs, a few of them present altered levels in cardiovascular diseases and diabetes mellitus [6, 7]. Notably circulating levels of miR-126 levels are importantly reduced in subjects with impaired glucose tolerance or type 2 diabetes [8]. In contrast, Let-7e [9] and miR-145 [10, 11] are importantly increased in different vascular pathologies. Additionally an important role for miR-33b [12] and miR-132 [13] have been suggested in the development of obesity due to their participation in lipid and cholesterol metabolisms as well as significant expression in adipose tissue [14]. In order to determine whether MetS components are associated with altered levels of MetS-associated miRNAs, we determined the plasma levels of Let-7e, miR-33b, miR-126, miR-132 and miR-145 in 126 children with one or more MetS trait and 30 control subjects.

## Methods

### Ethics statement

This study was conducted according to the principles expressed in the “Declaration of Helsinki”. Parents or children representatives signed an informed consent form, and boys/girls an informed acceptance form. The study was approved by the Ethic committee of the School of Medicine of the Pontificia Universidad Católica de Chile, and Fondo Nacional de Desarrollo Científico y Tegnológico de Chile (FONDECYT, Chile).

### Sample

The sample selected was part of a larger study of cardiovascular risk factors in children 10 to 14 years. Briefly, in a first stage the original study evaluated the number of MS components for 3329 children among other measurements. In a second stage, the students who presented certain pre-established combinations of MS components were eligible to conform groups to apply more specific measurements; about 20% of the cases were normal in their nutritional diagnoses and were included as controls.

These two stages were successively performed during a 3 year period. Plasma samples from the participants of the last year were used to determine the levels of 4 microRNAs: miRNA Let-7e, miRNA 126, miRNA 132, and miRNA145. The samples were processed blinded to the knowledge of the nutritional status or the cardiovascular risk factors of the studied subjects.

### Subjects

This study considered a subgroup of 156 subjects (age 10 to 12 y.o., 90 females and 66 males) conveniently selected due to the presence of a combination of MetS traits (presence of altered lipids and glucose plasma levels, central obesity and elevated blood pressure according to Cook´s criteria) and a control group without any MetS trait, from a retrospective non concurrent cohort study of school-age children from 20 public schools (total number 3325) [15] managed by the Municipality of Puente Alto, Santiago, Chile. Groups analyzed included 30 control subjects with no MetS traits as described below, and subjects with one (n = 50), two (n = 41), and three or more (n = 35) MetS traits. Socio-economic status was estimated using as a proxy the number of maternal years of formal education.

### Metabolic syndrome criteria

The criteria of Cook et al. were used to define MS in the studied children [4], when at least three out of five of its components were present, as defined by the following cut-off points: waist circumference (WC) ≥ 90^th^ percentile; blood pressure (BP), either systolic (SBP) or diastolic (DBP), ≥ 90^th^ percentile, low high density lipoprotein cholesterol (HDL-C) ≤ 40 mg/dL; triglycerides (TG) > 110 mg/dL, and glycaemia (GLU) ≥100 mg/ dL. The evaluation at each school was made by a trained Nurse and a Nutritionist. Height and weight were measured using a stadiometer and a beam-scale Seca, with an accuracy of 50 g, while being barefoot and lightly clothed. The final height and weight were the respective averages of three measurements; in the latter, the average weight of their clothes was deducted. Waist circumference was measured with inextensible tape on the upper lateral border of the right ilium in the mid-axillary line at the end of an exhalation; three measurements were averaged and we used ≥ 90th percentile as cut-off value [16]. A blood pressure monitor (Critikon Dinamap Pro 100) was used according to international norms and the averages of three measurements of SBP and DBP were obtained and classified as abnormal using the ≥ 90 percentile of the same reference [17]. A voluntary private self-report of pubertal status was requested by observation of standardized photos of breast development in girls and genitalia in boys, including the presence of pubic hair. All cut-off values applied to the subjects in this study were referred to curves generated from the whole cohort previously studied.

### Blood sample analysis

Venous blood samples under fasting conditions were collected for determination of glucose (Gluco-quant method, Glucose / Hexokinase, Roche Diagnostics GmbH, Mannheim) and insulin (immunoassay direct luminometer chemotherapy, ADVIA Centaur XP. Bayer HealthCare LLC, Kyowa Medex Co, Japan), this method measures concentrations of insulin from 0.5 to 300 mUI / mL (sensitivity 0.5 mUI / mL) with a coefficient of variation of 3.48% and 6.17% for concentrations of 23.51 mUI / mL and 62.49 mUI / mL, respectively. HOMA was calculated using the formula [(Glucose (mmol / L) x insulinaemia (mUI / mL)) / 22.5]. Triglycerides and HDL-C were analyzed on the Modular Analytics P-800 platform (Roche Diagnostics GmbH, Mannheim, Germany).

### RNA extraction

Plasma samples for micro-RNA determination and analysis were conducted as doubled blinded to prevent biased results. Total RNA was extracted from 250 μL of plasma of supernatant fraction according to the supplier instructions (miRNAesy extraction kit, Qiagen). For normalization, 250 pmol of syn-cel-miR-39 miScript miRNA Mimic (Qiagen) were added to the supernatant 1 fraction just before the denaturation step from the organic extraction [10].

### Reverse transcription

Sequence-specific reverse transcription of hsa-miR-33b, hsa-miR-126, hsa-miR-132, hsa-miR-145, hsa-Let-7e and Cel-miR-39 were achieved with TaqMan microRNA Reverse Transcription kit (Applied Biosystem).

### Preamplification and amplification

A pre-amplification step was carried out before the qPCR analysis, using TaqMan PreAmp Master Mix and TaqMan Universal PCR Master Mix II, respectively (Applied Biosystem). Relative quantity was calculated by mean 2-ΔΔCt method regarding the synthetic control syn-cel-miR-39.

### Let7e and miR-126 targets prediction and interaction

Prediction of targets for Let-7e and miR-126 were independently analyzed using the previously validated web software TargetScan 6.2 (http://www.targetscan.org) [18, 19] and miRanda (http://www.microrna.org) [20]. Selection of targeted messenger RNAs was based on the presence of a low “context score” and a high “probability of conserved targeting” [19] and the coincidence between the two algorithms. Following, the interaction of the proteins which mRNA are targeted by the analyzed miRNAs were evaluated with the web software String 9.1 (http://string-db.org/) [21, 22] using the highest confidence score (0.900) for protein-protein interaction, a maximum of 20 interactors and excluding all the proteins not regulated by the miRNAs analyzed.

### Statistics

Differences in anthropometry (WC, waist to hip ratio, BMI), blood sample (TG, VLDL, HDL, glycaemia, insulin, HOMA-IR) and miRNA (Let7e, 126, 132 and 145) averages by number of MS components were evaluated using one way ANOVA for independent samples. Post hoc comparisons were assessed using Tamane or Bonferroni correction. Additionally, linear association between continuous variables was evaluated using Pearson’s correlation test. To describe a possible additive effect of each MS component, ANOVA test for independent samples and its post hoc test was used between miRNA averages for 3 groups: without a specific MS component, with only the specific MS component and with the MS component and at least one more. To evaluate the diagnostic accuracy, we generated receiver operating characteristic (ROC) curve for the different miRNA studied. The sensitivity and specificity which maximized the area under the ROC curve (AUC) were used to assess the diagnostic accuracy of miRNA in cases and controls. For all analysis SPSS 17.0 was used. Significant P-value was defined as <0.05.

## Results

### Characteristics of selected children

**Fig 1:**
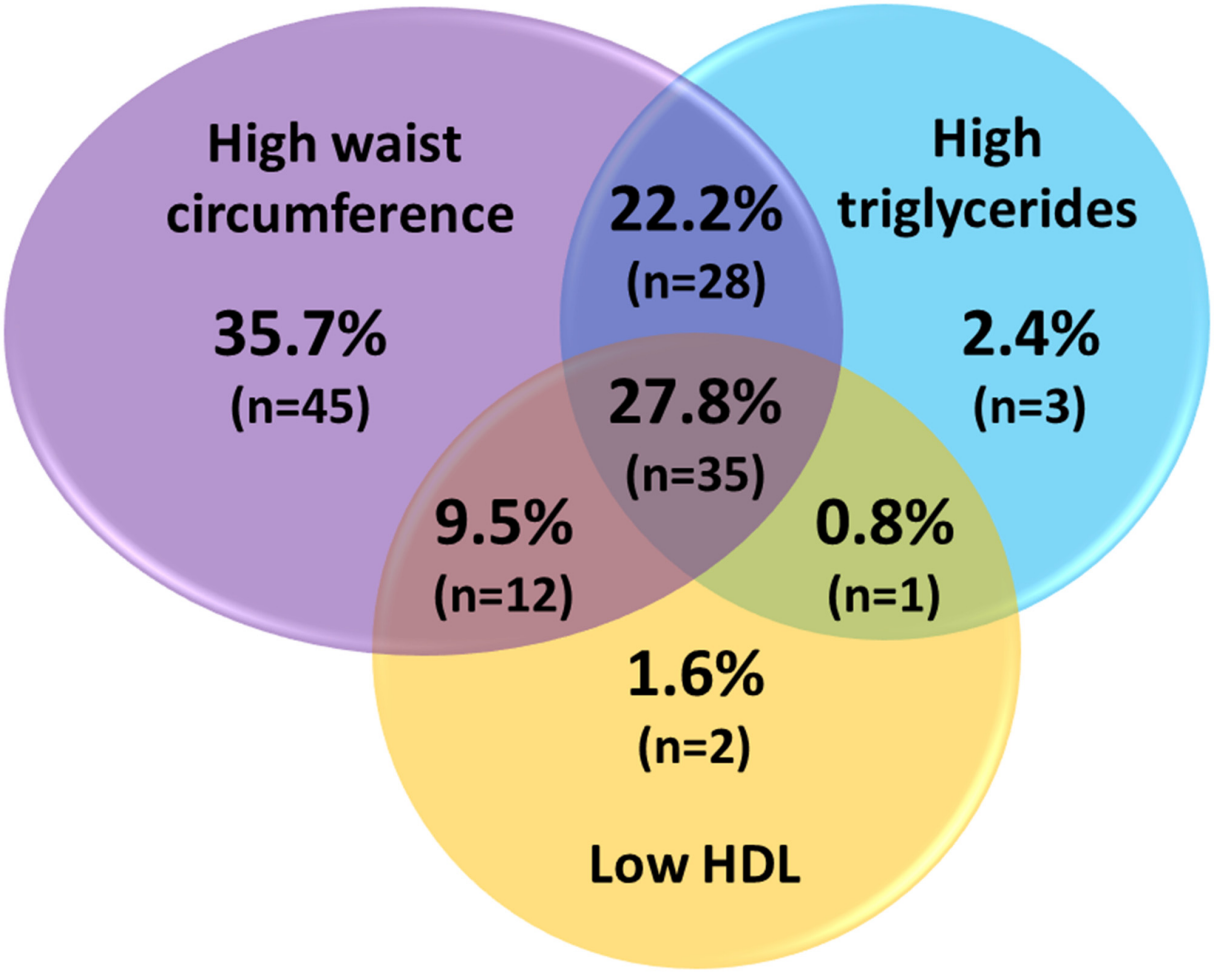
Presence of different MetS traits in the children with cardiometabolic risk. Venn diagram showing the percentage and total number (in parenthesis) of children with increased waits circumference, increased triglycerides and decreased HDL plasma levels alone and their combinations in the groups studied.

A comparable number of subjects (30–50) without or with one, two or three components of MetS were selected from a larger cohort. General characteristics of the children studied grouped by gender showed differences between girls and boys pubertal development, presence of overweight and altered triglycerides levels which were higher in girls than boys (Table 1). Additionally the presence of obesity and altered HOMA-IR were higher in the groups of boys compared to girls. Among the selected children with one or more MetS-related alterations, increased waist circumference as a single alteration was present in 45 of 50 subjects who showed 1 MetS trait (Fig 1). Conversely, altered plasma lipid levels (i.e. high triglycerides or low HDL) as a single MetS trait showed a low presence (5 of 50 subjects) and were mainly associated with increased waist circumference (75 of 126 showed high waist circumference together with altered lipid levels).

**Table 1:** General characteristics of the study group according to sex.

	Boys		Girls		Total		P-value
	N = 66		N = 92		N = 158		
Age (years, mean [± SD])	11.63	[0.92]	11.65	[0.90]	11.64	[0.90]	*0*.*920*
Pubertal (Tanner II-V, n [%])	54	[81.8]	91	[98.9]	145	[91.8]	*<0*.001*
Weight (kg, mean [± SD])	57.3	[12.1]	55.3	[11.4]	56.2	[11.7]	*0*.*285*
Height (cm, mean [± SD])	151	[7.4]	150	[6.3]	150.4	[6.8]	*0*.*337*
BMI (mean [± SD])	24.9	[4.0]	24.4	[4.0]	24.6	[4.0]	*0*.*432*
z-BMI (mean [± SD])	1.6	[0.7]	1.4	[0.8]	1.5	[0.8]	*0*.*079*
Obesity (n [%])	46	[71.9]	41	[44.6]	87	[55.8]	*0*.001*
Overweight (n [%])	10	[15.6]	34	[37.0]	44	[28.2]	*0*.004*
WC ≥ 90^th^ percentile (n [%])	52	[81.3]	68	[73.9]	120	[76.9]	*0*.*285*
Triglycerides ≥ 110 mg/dL (n [%])	21	[32.8]	46	[50.0]	67	[42.9]	*0*.033*
HDL ≤ 40 mg/dL (n [%])	16	[25.0]	34	[37.0]	50	[32.1]	*0*.*115*
HOMA-IR ≥ 90^th^ percentile (n [%])	20	[30.3]	15	[16.7]	35	[22.4]	*0*.044*
MetS ≥3 components (n [%])	10	[15.6]	25	[27.2]	35	[22.4]	*0*.*089*
RQ Let-7e (mean [± SD])	2.37	[4.15]	2.57	[4.41]	2.49	[4.29]	*0*.*776*
RQ miR145 (mean [± SD])	1.57	[2.72]	1.89	[4.53]	1.74	[3.87]	*0*.*646*
RQ miR132 (mean [± SD])	2.27	[5.40]	1.56	[2.03]	1.86	[3.82]	*0*.*252*
RQ miR126 (mean [± SD])	1.78	[2.28]	2.07	[3.23]	1.95	[2.87]	*0*.*535*

WC, waist circumference; RQ, relative quantification.

**Fig 2:**
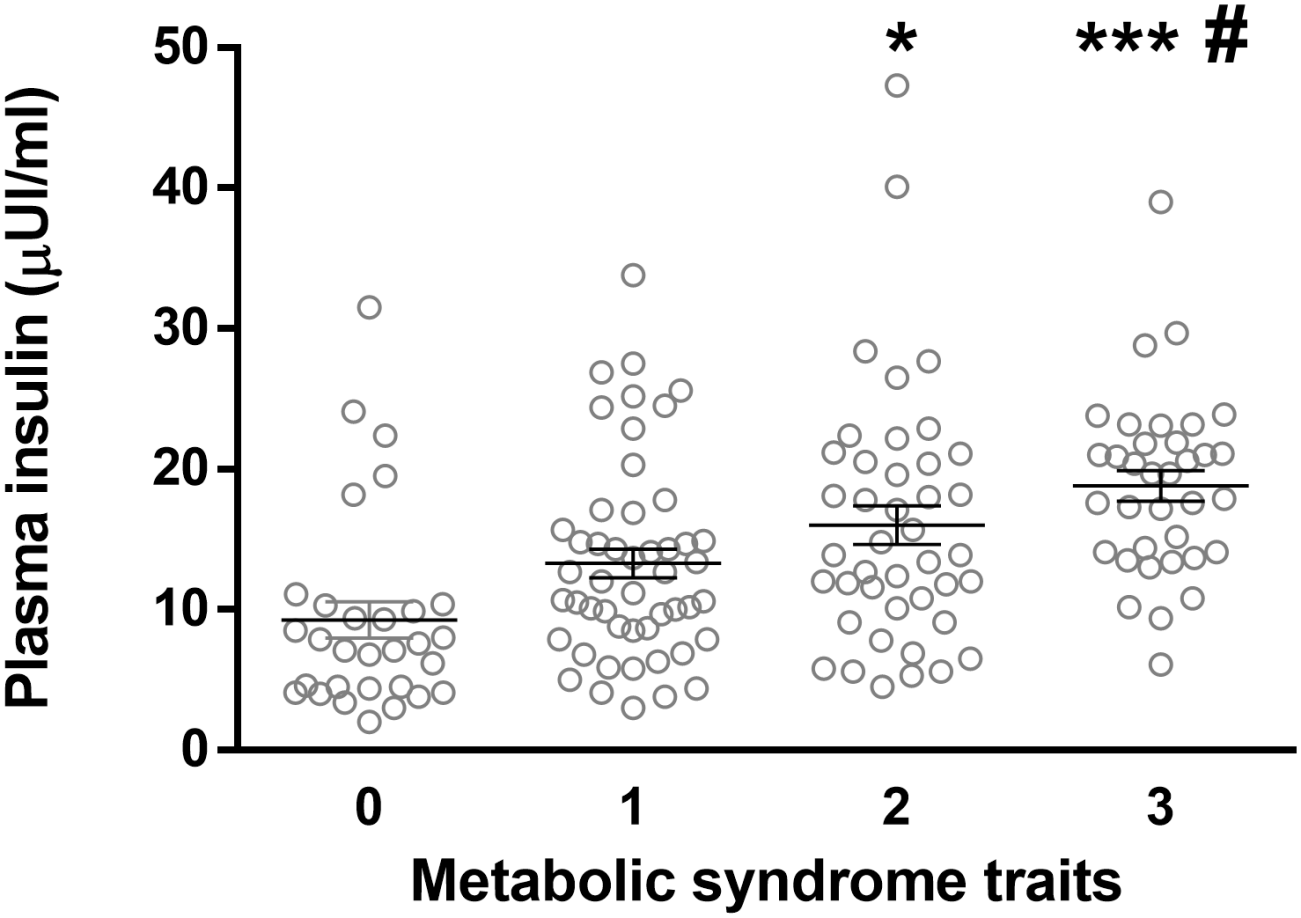
Insulinaemia in children with and without MetS traits. Insulinaemia in children without (0) and with one (1), two (2) or three (3) MetS traits. Values are mean ± SEM, *p < 0.05 and ***p < 0.001 vs. controls, and ^#^ *p* < 0.05 vs. one MetS trait, ANOVA.

HDL-C in subjects with one or two MS traits were over the cut-off used (≤ 40 mg/dL) but significantly decreased (12% and 20%, respectively) compared with control subjects, furthermore subjects with two MetS present lower HDL-C levels than subjects with one trait. On the other hand, the presence of one or more MetS traits was associated with increased waist to hip ratio (~1.1 fold), body weight (~1.3 fold) as well as body mass index (~1.3 fold) (S1 Table). Among the subjects studied there were no differences in blood pressure (i.e. mean, systolic or diastolic) and fasting glycaemia. In contrast, fasting insulin plasma levels were not different between subjects with one MetS trait and controls, but increased in subjects with two (~1.7 fold) or three (~2.0 fold) MetS traits (Fig 2), and this was also observed for HOMA-IR index (data not shown).

### Metabolic syndrome traits and micro-RNAs plasma levels

Four out of five miRNAs considered in this study were detected in plasma samples from children with or without MS traits. Namely Let-7e, miR-126, miR-132 and miR-145 were present, whilst miR-33b was not detectable in plasma, however it was found in a control sample of miRNAs from HEK-293 cells as reported (S1 Fig) [23, 24]. Mean plasma levels of Let-7e, miR-126, miR-132 and miR-145 were not different between female and male subjects (Table 1). Regression analysis of the different MetS traits analyzed as continuous variables regarding the different miRNAs analyzed (Table 2) showed that Let-7e presented a negative association with HDL-C levels, but a positive correlation with the number of MetS traits. Levels of miR-126 presented a positive correlation with waist circumference, waist to hip ratio, BMI, and plasma triglycerides and VLDL-C. Additionally plasma levels of miR-132 showed a positive correlation with waist to hip ratio. On the other hand, miR-145 did not show significant correlation with any trait determined (S2 Table). Similarly there were no correlation between body weight, insulinaemia, HOMA-IR and blood pressure with miRNAs analyzed (S2 Table).

**Table 2:** Significant correlations of different MetS traits with plasma levels of selected miRNAs.

		Let-7e	miR-126	miR-132
**Waist circumference (cm)**	*Correlation coefficient*		**0.210**	
	*p*		***0.008***	
**Waist to hip ratio (cm/cm)**	*Correlation coefficient*		**0.220**	**0.183**
	*p*		***0.006***	*0*.*021*
**BMI (kg/m** ^**2**^ **)**	*Correlation coefficient*		**0.185**	
	*p*		***0.020***	
**Triglycerides (mg/dL)**	*Correlation coefficient*		**0.175**	
	*p*		***0.028***	
**HDL (mg/dL)**	*Correlation coefficient*	**-0.168**		
	*p*	***0.035***		
**VLDL (mg/dL)**	*Correlation coefficient*		**0.173**	
	*p*		*0*.*030*	
**Number of MetS traits**	*Correlation coefficient*	**0.255**		
	*p*	*0*.*001*		

**Fig 3:**
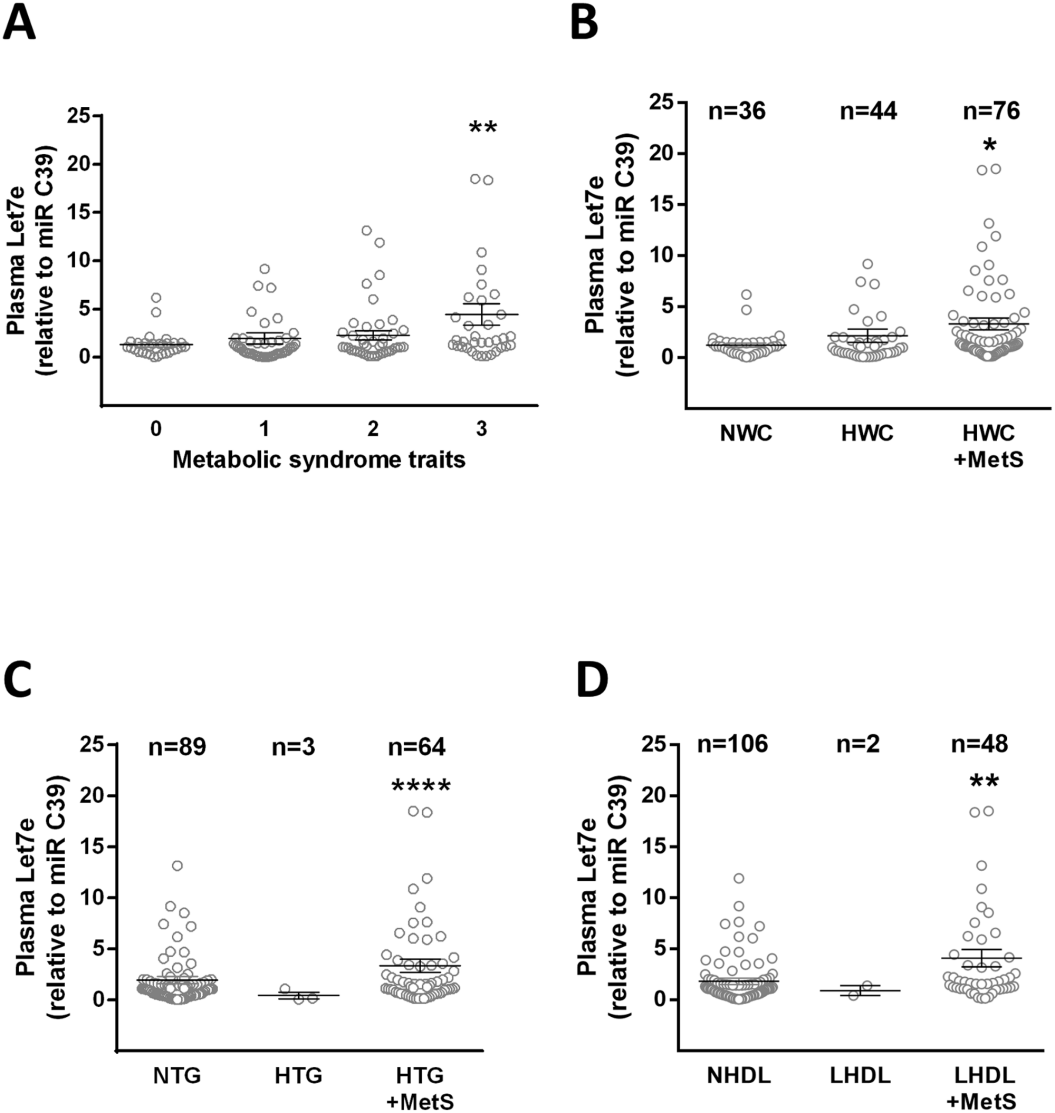
Plasma levels of Let-7e according to the presence of MetS traits. (A) Plasma levels of Let-7e according to the number of MetS traits, or (B) the presence of altered waist circumference (HWC), (C) elevated plasma triglycerides (HTG) and (D) low HDL plasma levels (LHDL) in combination with one other MetS trait (+MetS). Values are mean ± SEM, * *p* < 0.05, **p < 0.01, ****p < 0.0001 vs. controls, ANOVA.

Comparison of Let-7e mean plasma levels according to the number of MetS traits showed an increased in subjects with three MetS traits (~4.5 fold) compared with control subjects (Fig 3A). Additionally Let-7e levels were compared in children with normal or altered waist circumference, high triglycerides and/or low HDL, independent of the presence of other MetS traits. Levels of Let-7e were not altered in subjects that only presented increased waist circumference compared with subjects with normal waist circumference, however were augmented (~3.3 fold) by the coexistence with any other MetS trait (Fig 3B). This was also observed in subjects with altered triglycerides or HDL levels (Fig 3C and 3D, respectively) where the addition of another MetS trait associated with increased levels of Let-7e. In contrast plasma levels of miR-126, miR-132 and miR-145 where not different among groups analyzed using this approach (data not shown).

**Fig 4:**
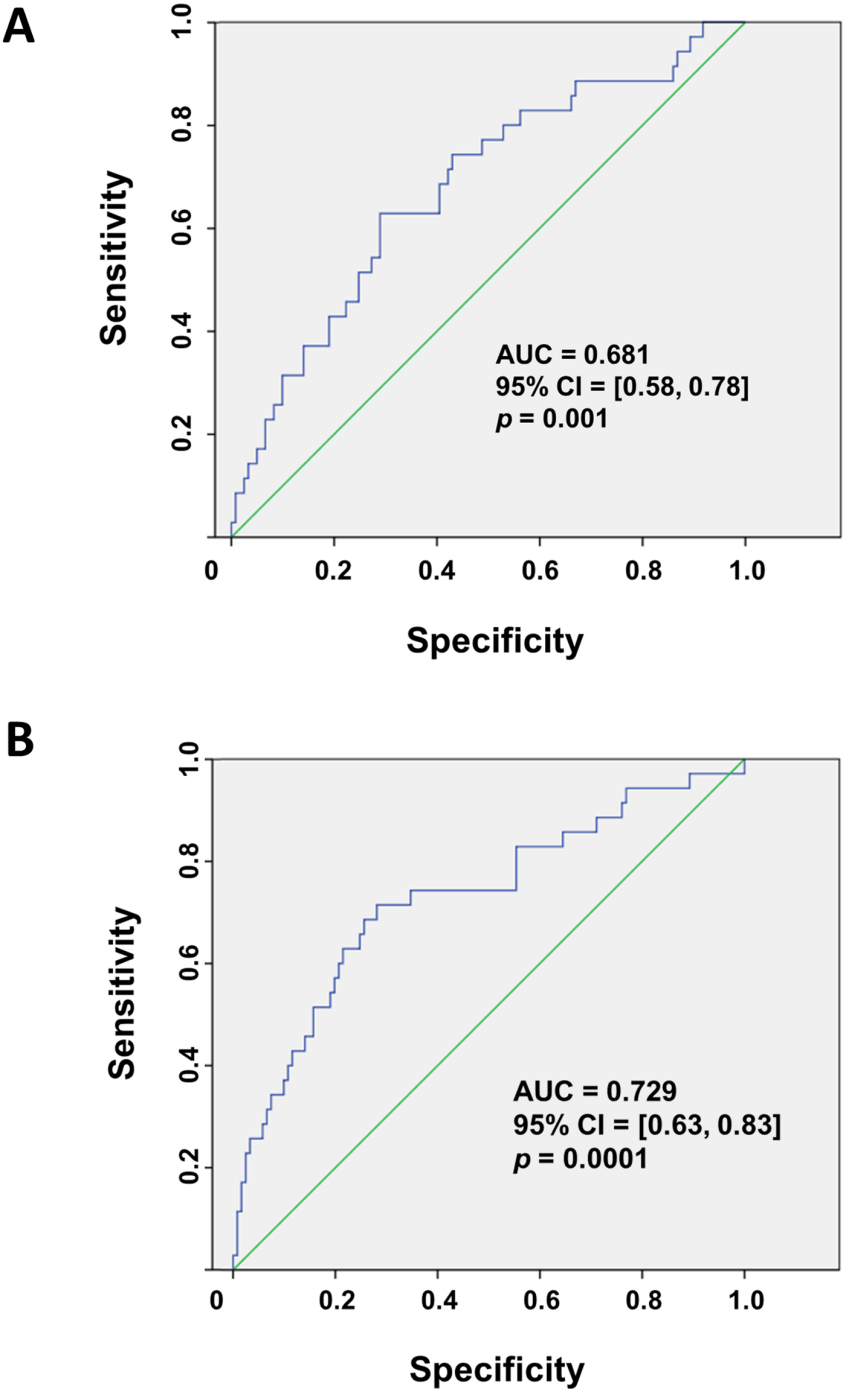
Operating characteristic (ROC) curve for Let-7e and the combination with miR-126. (A) ROC curve for plasma levels of Let-7e alone or (B) in combination with levels of miR-126 in pediatric subjects with and without metabolic syndrome according to Cook´s criteria.

Further analysis of ROC curves to evaluate the potential diagnostic value of Let-7e and miR-126 levels for metabolic syndrome in children showed that Let-7e levels higher than 1.52 RQ presented a significant AUC (0.681) with a sensitivity of 62.9% and a specificity of 71.1% (Fig 4A), whilst miR-126 did not show a significant value (data not shown). However when RQs for Let-7e and miR-126 were considered together there was an improvement in the sensitivity (71.4%) and AUC (0.729) without changes in the specificity (71.9%) (Fig 4B).

### *In silico* analysis of potential pathways compromised

**Fig 5:**
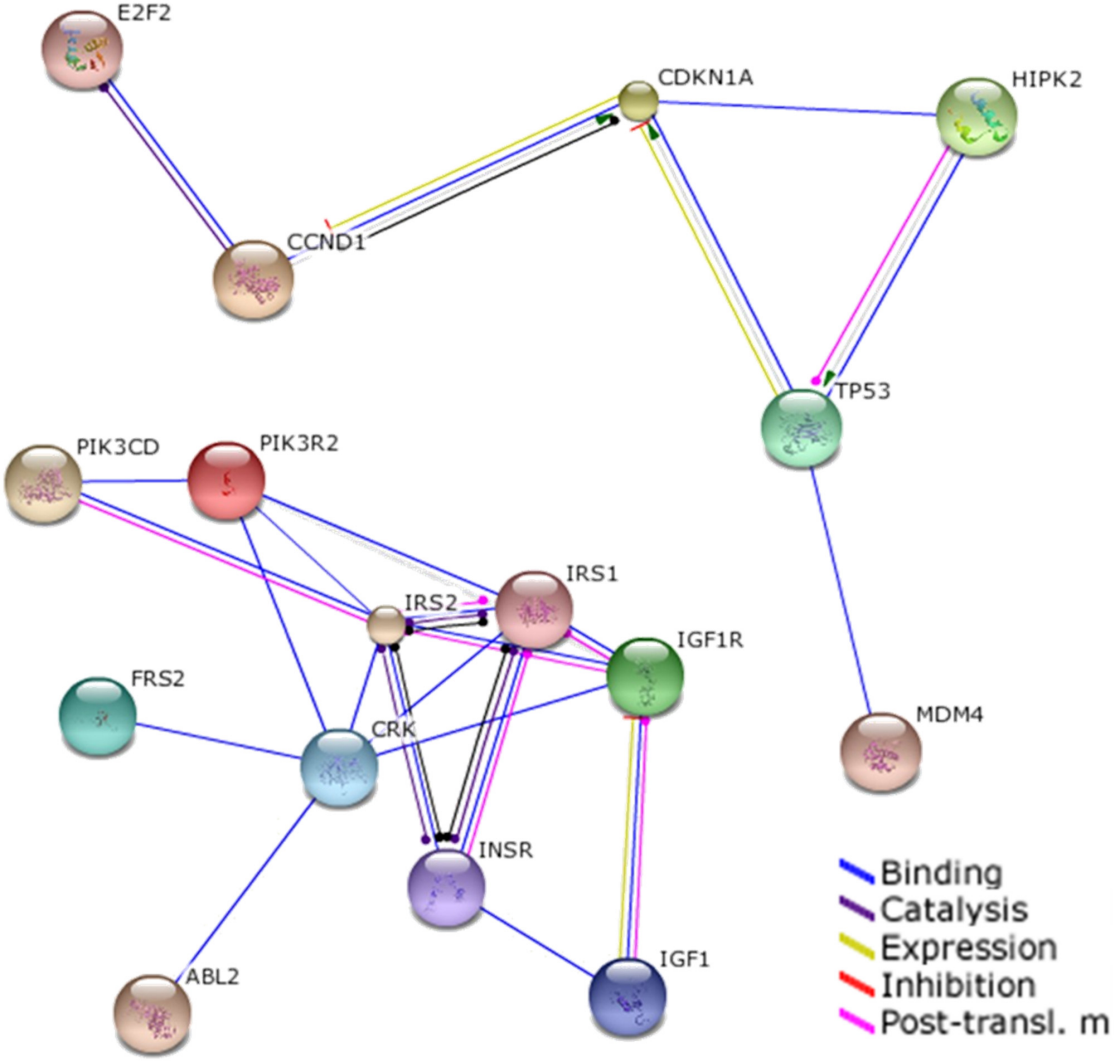
Convergence of Let-7e and miR-126-regulated signalling pathways at protein level. *In silico* analysis of the interactions of proteins derived from mRNA targeted by Let-7e and miR-126. Interacting proteins are linked by colored lines which denote binding (blue), catalytic effect (purple), expression regulation (yellow), inhibition (red end) and post-translational modification (fuchsia).

Since plasma levels of miR-126 showed a significant positive correlation with several MetS traits, and Let-7e was increased in subjects with increased waist circumference along with other trait risk, an *in silico* analysis searching potential targets for both miRNAs and their interactions was performed using two different algorithms. Analysis for potential targets of Let-7 and miR-126 in human showed 340 mRNA targets for Let-7, and 102 targets for miR-126 of which 46 were targeted by both miRNAs. In contrast interaction analysis using the highest confidence score values showed that 15 of 442 proteins (~3%) derived from these mRNA were potential interacting targets (Fig 5) which were mainly related with cell cycle, apoptosis, growth factors and metabolism (Table 3). Notably, most of these proteins are involved in the signaling pathway of insulin and IGF1 (Fig 5).

**Table 3:** RNAs targeted by Let-7 and miR-126 whose proteins present functional interactions.

Protein	Name	Function	Targeted by
ABL2	Abelson tyrosine-protein kinase 2	Cytoskeleton, cell motility	Let-7
CCND1	Cyclin D1	Cell cycle/apoptosis	Let-7
CDKN1A	Cyclin-dependent kinase inhibitor 1A	Cell cycle/apoptosis	Let-7
CRK	Adapter molecule crk	Cell cycle/apoptosis	miR-126
E2F2	E2F transcription factor 2	Cell cycle/apoptosis	Let-7
FRS2	Fibroblast growth factor receptor substrate 2	Growth factor/metabolism	Let-7
HIPK2	Homeodomain interacting protein kinase 2	Cell cycle/apoptosis	Let-7
IGF1	Insulin-like growth factor 1	Growth factor/metabolism	Let-7
IGF1R	Insulin-like growth factor 1 receptor	Growth factor/metabolism	Let-7
INSR	Insulin receptor	Growth factor/metabolism	Let-7
IRS1	Insulin receptor substrate 1	Growth factor/metabolism	miR-126
IRS2	Insulin receptor substrate 2	Growth factor/metabolism	Let-7/miR-126
MDM4	Mdm4 p53 binding protein homolog	Cell cycle/apoptosis	Let-7/miR-126
PIK3CD	Phosphatidylinositol-4,5-bisphosphate 3-kinase, catalytic subunit delta	Cell signaling kinase/phosphatase	miR-126
PIK3R2	Phosphoinositide-3-kinase, regulatory subunit 2 (beta)	Cell signaling kinase/phosphatase	Let-7/miR-126
TP53	Tumor protein p53	Cell cycle/apoptosis	Let-7

**Fig 6:**
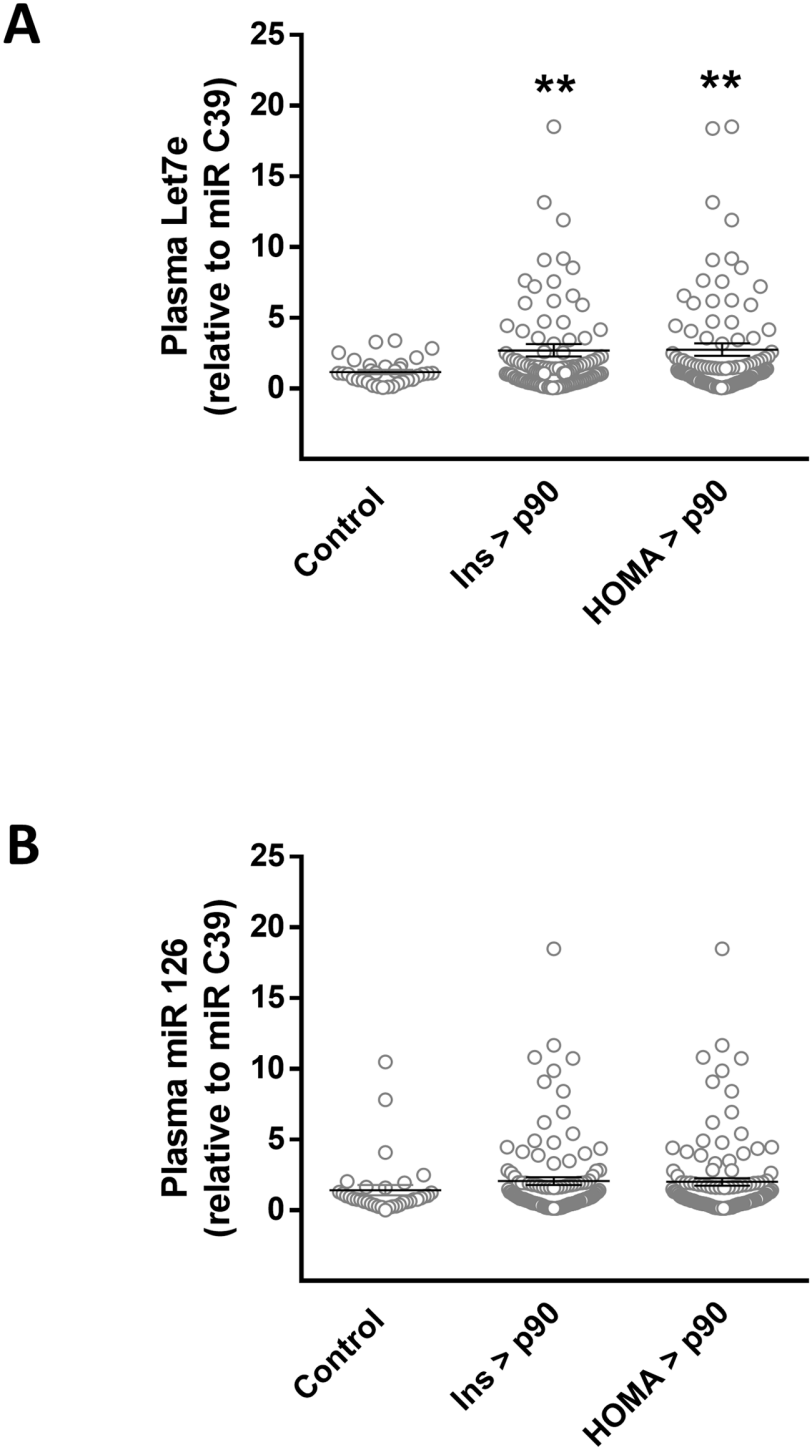
Circulating levels of Let-7e and miR-126 and insulin resistance. (A) Plasma levels of Let-7e and (B) miR-126 in controls (open bars) and children with insulinaemia (light grey bars) and HOMA-IR (solid bars) over 90th percentile (light grey bars). Values are mean ± SEM. **p < 0.01. T-test.

To address whether circulating Let-7e and miR-126 associated with markers of insulin resistance, plasma levels of both miRNAs were compared between controls and children with altered insulinaemia and HOMA-IR (over 90^th^ percentile regarding the whole cohort previously studied [15]). Subjects with increased insulinaemia and HOMA-IR showed a ~2 fold increase in Let-7e levels compared with control (Fig 6A), without significant changes in miR-126 (*p* = 0.118 for insulin, *p* = 0.158 for HOMA-IR, versus control) (Fig 6B).

## Discussion

This study demonstrates for the first time in pediatric subjects that MetS components are associated with increased plasma levels of miRNAs that have been associated to MetS in adults. These results were obtained in a selected pediatric population, from a larger cohort, in which central obesity was the main parameter altered along with elevated insulinaemia and HOMA-IR. The analysis of continuous variables demonstrated that plasma levels of miR-126 significantly correlates with waist circumference, waist to hip ratio, BMI, plasma triglycerides and VLDL. This is also observed for miR-132 regarding waist to hip ratio. On the other hand Let-7e plasma levels showed a significant increase in subjects with three MetS traits, and positively correlated with the number of MetS traits present. Increased levels of Let-7e occurred mainly in subjects that present increased waist circumference along with one other altered parameter. Evaluation of Let-7e and miR-126 ROC curves as markers of metabolic syndrome in children showed that only Let-7e plasma levels presented a potential predictive value which was improved when miR-126 levels were considered. Finally, *in silico* analysis of the potential targets of the most significantly altered miRNAs (i.e. Let-7e and miR-126) showed that both converge in regulating the insulin signaling pathway, which connects with the increased insulinaemia and HOMA index present in these subjects.

Metabolic syndrome along with the individual cardiometabolic conditions associated to it, represent the most prevalent diseases in adults [25], however growing evidence shows that the presence of MS traits in children and adolescents associates to increased risk of developing cardiometabolic diseases in adult life [26]. There is no consensus regarding the diagnose of MS in pediatric population, mainly due to the absence of clear cut-off values for the classical parameters used for its diagnosis in adults [1, 3]. In this study, using Cook´s et al. criteria, we evaluated in children the association of different MS traits with plasma levels of miRNAs that have been reported altered in adults with cardiometabolic dysfunction. In order to provide insight on new markers associated to the risk of developing MS, we aimed to determine the plasma levels of specific miRNA in children with altered cardiometabolic traits. The strength of plasma miRNAs as molecular markers relies on several characteristics of these molecules. First, the stability of miRNAs in plasma is comparable to proteins, and higher than mRNAs, which in combination with the use of quantitative PCR on their detection rises the possibility to measure small changes in their levels with higher precision than proteins and other biomarkers [5, 6]. At cellular level it is known that a single miRNA can control the expression of several mRNA and the transcriptional activity of genes [19, 27], which in most of the cases affects the gene expression of specific molecular pathways [28]. Additionally part of plasma miRNAs circulate associated to vesicles with potential endocrine effects [29, 30] which is nowadays under study [5]. In spite of the fact that the precise role of circulating miRNAs is unknown, evidence shows that their levels are altered in several pathological conditions, and they change in response to pharmacological treatments earlier than routine clinical markers [31, 32]. These characteristics have led to propose the use of circulating miRNA as molecular markers in cancer, metabolic disorders and cardiovascular diseases [5, 6, 33].

Studies characterizing the profile of circulating miRNAs show that among hundreds of miRNAs normally present in plasma, there is a small proportion of them altered in adults with cardiovascular and metabolic diseases [8–11, 34, 35]. In fact a recent review shows that Let-7e and miR-126 along with other 6 miRNAs are among the most prominent miRNAs implicated in cardiometabolic disorders [36]. Moreover little is known about the levels of miRNAs in normal children, and especially those that have been highlighted as markers of cardiometabolic dysfunction in adults. In order to determine whether some miRNAs reported as altered in adults with MS-related diseases we determined the levels of Let-7e, miR-33b, miR-126, miR132 and miR-145.

Results show that plasma levels of Let-7e were increased in children with three MS traits, and positively correlated with the number of traits. Conversely mean plasma levels of miR-126, miR-132 and miR-145 were unchanged in children grouped according to the number of MS traits. Despite that miR-126 has been reported decreased in vascular diseases [6] and the fact that the subjects recruited in this study did not present vascular alterations (i.e. increased blood pressure), we found that miR-126 positively correlated with several metabolic traits (i.e. waist circumference, BMI and triglycerides). On the other hand an important role for miR-33b has been suggested in the development of obesity due to its participation in lipid and cholesterol metabolism and is highly expressed in adipose tissue [14], however miR-33b was not detectable in plasma samples from children as reported for adults [24].

Considering that Let-7e and miR-126 showed important associations with MS traits in the children studied, an *in silico* analysis was performed to identify common potential targets regulated by both miRNAs. Notably Let-7e and miR-126 showed genes related with insulin signaling pathway as common targets, which could reflect the higher insulinaemia and HOMA-IR present in children with two or more MS traits. In this context, circulating levels of Let-7e were importantly increased in subjects with altered insulinaemia and HOMA-IR as well as showed a significant diagnostic value in ROC analysis, whilst there was a trend in miR-126 levels supporting the role of this miRNAs as a marker for insulin resistance in pediatric population. Conversely when plasma levels of both miRNAs were considered in the analysis, the predictive value of Let-7e was improved from less predictive to moderately predictive [37], suggesting that a potential use of these markers as diagnostic tool should include the levels of the two miRNAs in order to have a significant clinical value. Insulin plays a key role in different conditions associated with MS including glucose and triglyceride metabolism, and immune and vascular function [38]. In this context several studies have shown that Let-7 family regulates insulin-related genes in pancreas [39], adipocytes [40] and skeletal muscle [41]. Additionally Let-7 regulates the gene expression of inflammatory mediators in T-cells [42] and endothelium [43]. Interestingly Let-7 overexpression in mice induces insulin resistance repressing the expression of several mediators of insulin signaling pathway [41]. On the other hand compelling data show that miR-126 plays a key role regulating endothelial function [44, 45]. Notably it is reported in a cohort of 822 adults that plasma levels of miR-126 is decreased in subjects that present impaired glucose tolerance or type-2 diabetes [8]. These results contrast with those found in this study where miR-126 levels positively correlated with MS traits in children, suggesting that reduced miR-126 in adults with type 2 diabetes could represent a final stage of metabolic dysfunction, whilst in the early stages, during childhood, a compensatory increase in miR-126 would be observed.

The present data suggest that circulating levels of Let-7e and miR-126 associate with different traits of metabolic syndrome in children with a potential role as biomarkers. There are few reports showing levels of circulating miRNAs in children with or without altered metabolic parameters [46, 47]. In one of these studies the identification of differentially expressed miRNAs was based on array wide screening and further validation by qPCR utilizing endogenous miRNAs for quantification. None of the miRNAs reported as altered in these studies includes those founded in this work suggesting a possible bias in analyzing selected miRNAs. However a validation method for miRNAs levels is not well established, and there is no consensus regarding a set of endogenous miRNAs for quantitative normalization. In this context the use of an external miRNA for quantification of miRNAs (spike-in) in biological fluids represents at the moment the best choice [48]. Thus despite that the miRNAs studied here were selected from studies conducted in adults, the normalization procedure increases the reliability of the results, which along with ROC analysis argue for a potential application of these miRNAs (i.e. Let-7e and 126) as biomarkers of metabolic syndrome in children. Nowadays metabolic alterations in these patients are well characterized by accessible and direct measurements (i.e. waist circumference, waist to hip ratio and BMI) however circulating miRNAs point to veiled subclinical conditions. In this context pre-clinical data show that overexpression of Let-7 in mice leads to impaired glucose tolerance and insulin secretion [49], suggesting that increased levels of Let-7e could represent a basis rather a consequence of altered insulin response in these children. Considering that these results represent levels of specific miRNAs in a selected group of pediatric children from a carefully characterized cohort, its clinical significance will require further validation in a larger population.

In summary, this study demonstrates for the first time that children at age 10 to 12 y.o. with increased risk of MS do not present changes in plasma levels of miRNAs related with altered vascular (i.e. miR-145) and adipocyte (i.e. miR-132) functions. However they show increased plasma levels of Let-7e and miR-126, which could be actively participating in the development of insulin resistance, agreeing with the increased insulinaemia and HOMA-IR observed in these subjects.

## Supporting Information

S1 Fig Micro RNA-33b is undetectable in plasma from children at pre-pubertal age. Representative amplification plot for Cel-miR-39 in HEK-293 (a) and plasma (b), and Hsa-miR-33b in HEK-293 (c) and plasma showing that levels of the later miRNA were almost undetectable on the plasma samples studied.
(TIF)

S1 Table (DOCX)

S2 Table (DOCX)
