# Supplementary figures and images for: Micro-RNAs Let7e and 126 in Plasma as Markers of Metabolic Dysfunction in 10 to 12 Years Old Children
Source: PLoS One. 2015 Jun 5;10(6):e0128140. doi: 10.1371/journal.pone.0128140 (PMC4457533; doi:10.1371/journal.pone.0128140)

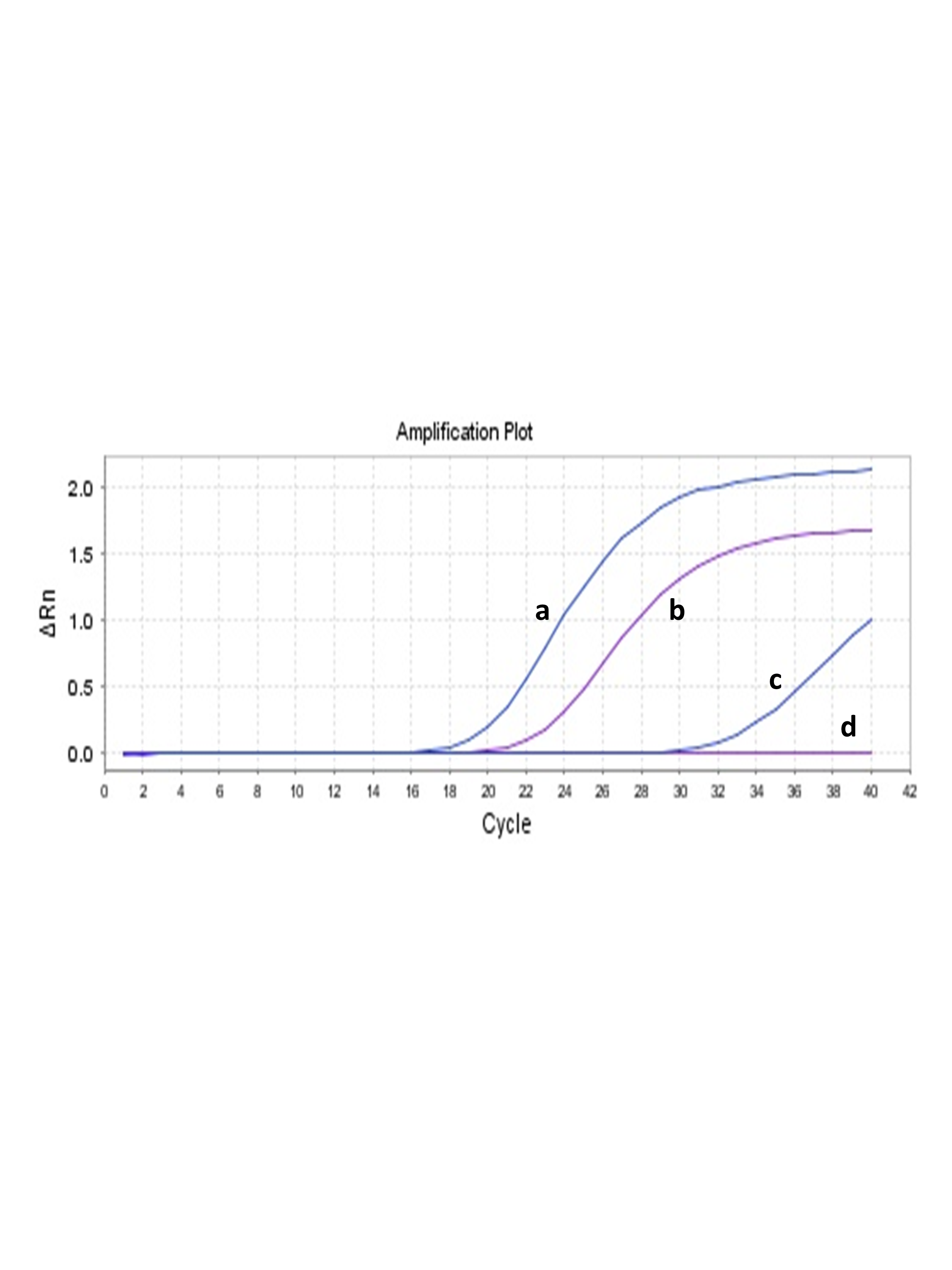

Supplement: S1 Fig — Representative amplification plot for Cel-miR-39 in HEK-293 (a) and plasma (b), and Hsa-miR-33b in HEK-293 (c) and plasma showing that levels of the later miRNA were almost undetectable on the plasma samples studied. (TIF) [file pone.0128140.s001.tif]
