# Supplementary material for: Micro-RNAs Let7e and 126 in Plasma as Markers of Metabolic Dysfunction in 10 to 12 Years Old Children
Source: PLoS One. 2015 Jun 5;10(6):e0128140. doi: 10.1371/journal.pone.0128140 (PMC4457533; doi:10.1371/journal.pone.0128140)
Supplement: S1 Table — (DOCX) [file pone.0128140.s002.docx]

**Supplementary Table 1. Metabolic markers in control and MetS groups.**

|  | **Control** | **1 MetS** | **2 MetS** | **3 MetS** |
| --- | --- | --- | --- | --- |
| **Waist circumference (cm)** | 73.3 ± 1.3 | 89.3 ± 1.2^a^ | 93.4 ± 1.2^a^ | 92.6 ± 1.1^a^ |
| **Waist to hip ratio (cm/cm)** | 0.89 ± 0.01 | 0.97 ± 0.01^a^ | 0.99 ± 0.01^a^ | 0.97 ± 0.01^a^ |
| **Body weight (kg)** | 43.7 ± 1.7 | 57.3 ± 1.4^a^ | 60.3 ± 1.6^a^ | 61.4 ± 1.6^a^ |
| **BMI (kg/m^2^)** | 19.5 ± 0.5 | 25.1 ± 0.4^a^ | 26.5 ± 0.5^a^ | 26.7 ± 0.5^a^ |
| **Triglycerides (mg/dL)** | 59.6 ± 3.9 | 73.8 ± 4.1 | 130.8 ± 8.2^a,b^ | 177.9 ± 9.6^a,b^ |
| **HDL (mg/dL)** | 55.6 ± 2.0 | 49.1 ± 0.9^c^ | 44.7 ± 1.1^a,d^ | 34.4 ± 0.9^a,b^ |
| **VLDL (mg/dL)** | 11.9 ± 0.8 | 14.7 ± 0.8 | 26.2 ± 1.6^a,b^ | 35.5 ± 1.9^a,b^ |

Values expressed as average ± SEM. ^a^ p < 0.0001 and ^c^ p < 0.05 vs control; ^b^ p < 0.0001 and ^d^ p < 0.05 vs 1 MetS.
