# Supplementary material for: Micro-RNAs Let7e and 126 in Plasma as Markers of Metabolic Dysfunction in 10 to 12 Years Old Children
Source: PLoS One. 2015 Jun 5;10(6):e0128140. doi: 10.1371/journal.pone.0128140 (PMC4457533; doi:10.1371/journal.pone.0128140)
Supplement: S2 Table — (DOCX) [file pone.0128140.s003.docx]

**Supplementary table 2. Non-significant correlations of different MetS traits with plasma levels of selected miRNAs**

|  |  | **Let-7e** | **miR-126** | **miR-132** | **miR-145** |
| --- | --- | --- | --- | --- | --- |
| **Waist circumference (cm)** | Correlation coefficient | 0.091 |  | 0.106 | 0.080 |
|  | *p* | *0.255* |  | *0.184* | *0.317* |
| **Waist to hip ratio (cm/cm)** | Correlation coefficient | 0.031 |  |  | 0.126 |
|  | *p* | *0.697* |  |  | *0.114* |
| **Body weight (kg)** | Correlation coefficient | 0.088 | 0.145 | 0.042 | 0.031 |
|  | *p* | *0.272* | *0.069* | *0.604* | *0.696* |
| **BMI (kg/m^2^)** | Correlation coefficient | 0.093 |  | 0.109 | 0.075 |
|  | *p* | *0.248* |  | *0.171* | *0.351* |
| **Triglycerides (mg/dL)** | Correlation coefficient | 0.127 |  | -0.025 | 0.090 |
|  | *p* | *0.111* |  | *0.752* | *0.261* |
| **HDL (mg/dL)** | Correlation coefficient |  | -0.070 | 0.013 | -0.005 |
|  | *p* |  | *0.381* | *0.868* | *0.950* |
| **VLDL (mg/dL)** | Correlation coefficient | 0.126 |  | -0.028 | 0.086 |
|  | *p* | *0.115* |  | *0.722* | *0.282* |
| **Insulin (µUI/mL)** | Correlation coefficient | 0.030 | -0.051 | -0.087 | -0.104 |
|  | *p* | *0.710* | *0.521* | *0.275* | *0.195* |
| **HOMA** | Correlation coefficient | -0.030 | -0.059 | -0.083 | -0.105 |
|  | *p* | *0.708* | *0.459* | *0.302* | *0.190* |
| **Mean blood pressure (mmHg)** | Correlation coefficient | 0.011 | 0.063 | 0.007 | -0.023 |
|  | *p* | *0.891* | *0.434* | *0.931* | *0.777* |
| **Diastolic blood pressure (mmHg)** | Correlation coefficient | 0.035 | 0.121 | 0.016 | 0.016 |
|  | *p* | *0.658* | *0.130* | *0.846* | *0.843* |
| **Systolic blood pressure (mmHg)** | Correlation coefficient | -0.049 | -0.100 | -0.016 | -0.095 |
|  | *p* | *0.542* | *0.212* | *0.841* | *0.235* |
